# Supplementary figures and images for: Epithelium intrinsic vitamin A signaling co-ordinates pathogen clearance in the gut via IL-18
Source: PLoS Pathog. 2020 Apr 24;16(4):e1008360. doi: 10.1371/journal.ppat.1008360 (PMC7202665; doi:10.1371/journal.ppat.1008360)

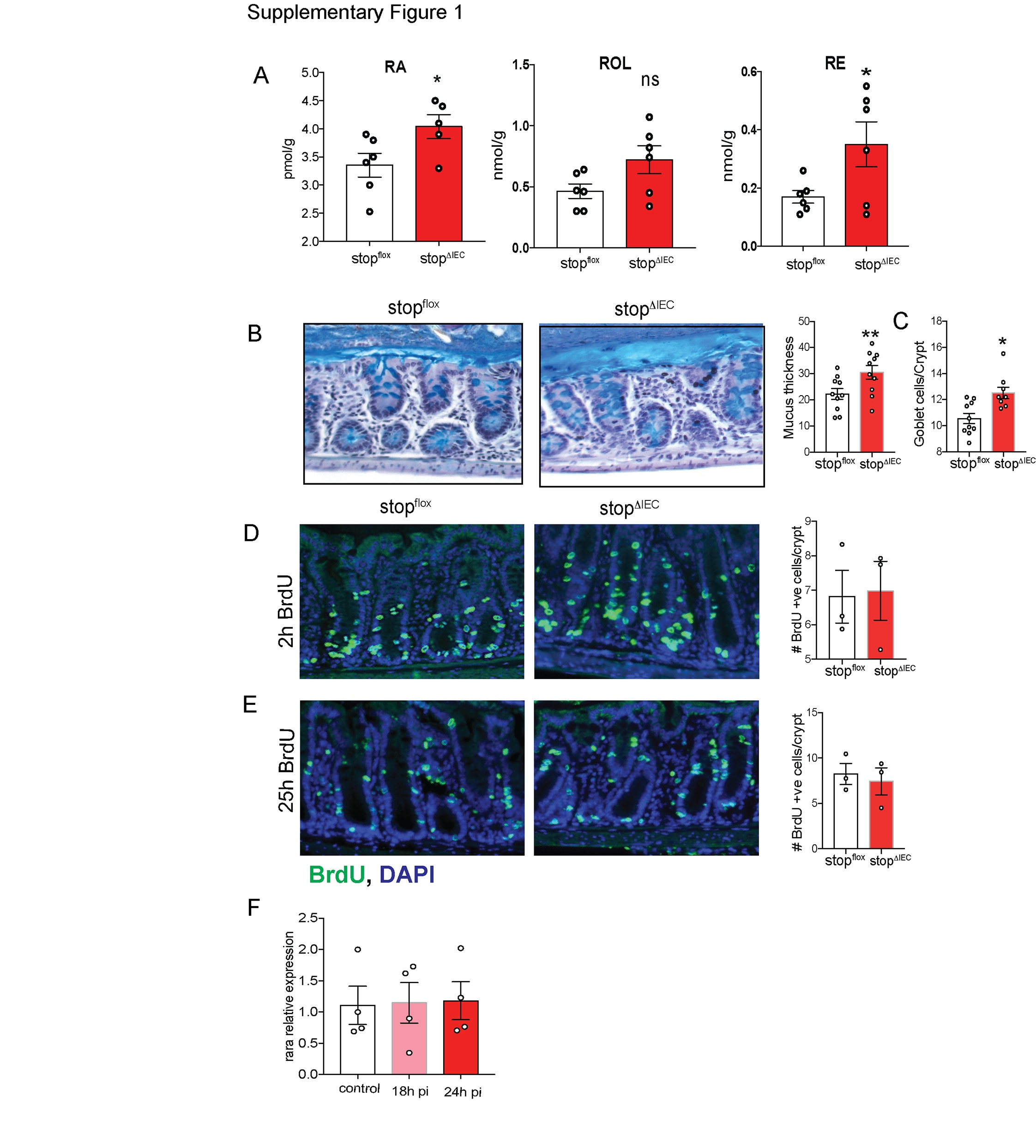

Supplement: S1 Fig — This figure compares the (A) Total RA (retinoic acid), ROL (retinol) and RE (retinyl ester) normalized per gram of colon tissue, (B) mucus thickness, (C) goblet cells/crypt, (D and E) epithelial turnover via BrdU incorporation at 2 h (D) and 25 h (E) post injection in homeostatic colons of stopflox and stopΔIEC mice. (F) Gene expression of retinoic acid receptor alpha in colon whole tissue at early and late timepoints of infection. (TIF) [file ppat.1008360.s001.tif]

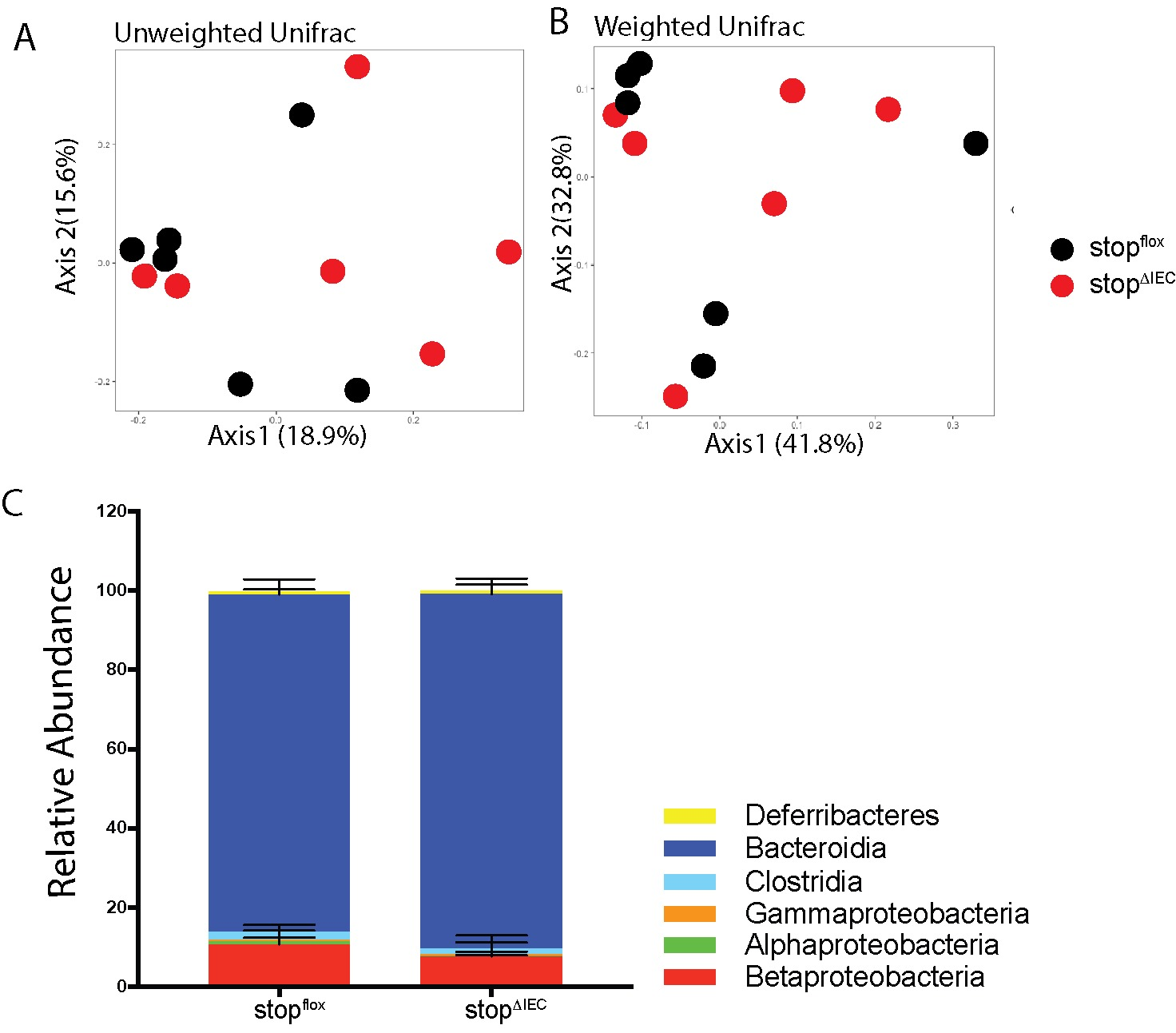

Supplement: S2 Fig — This figure analysis the fecal microbial communities in homeostatic stopflox and stopΔIEC mice using (A) unweighted Unifrac, (B) weighted Unifrac and displays the (C) relative abundance of microbial communities at the Class level. (TIF) [file ppat.1008360.s002.tif]

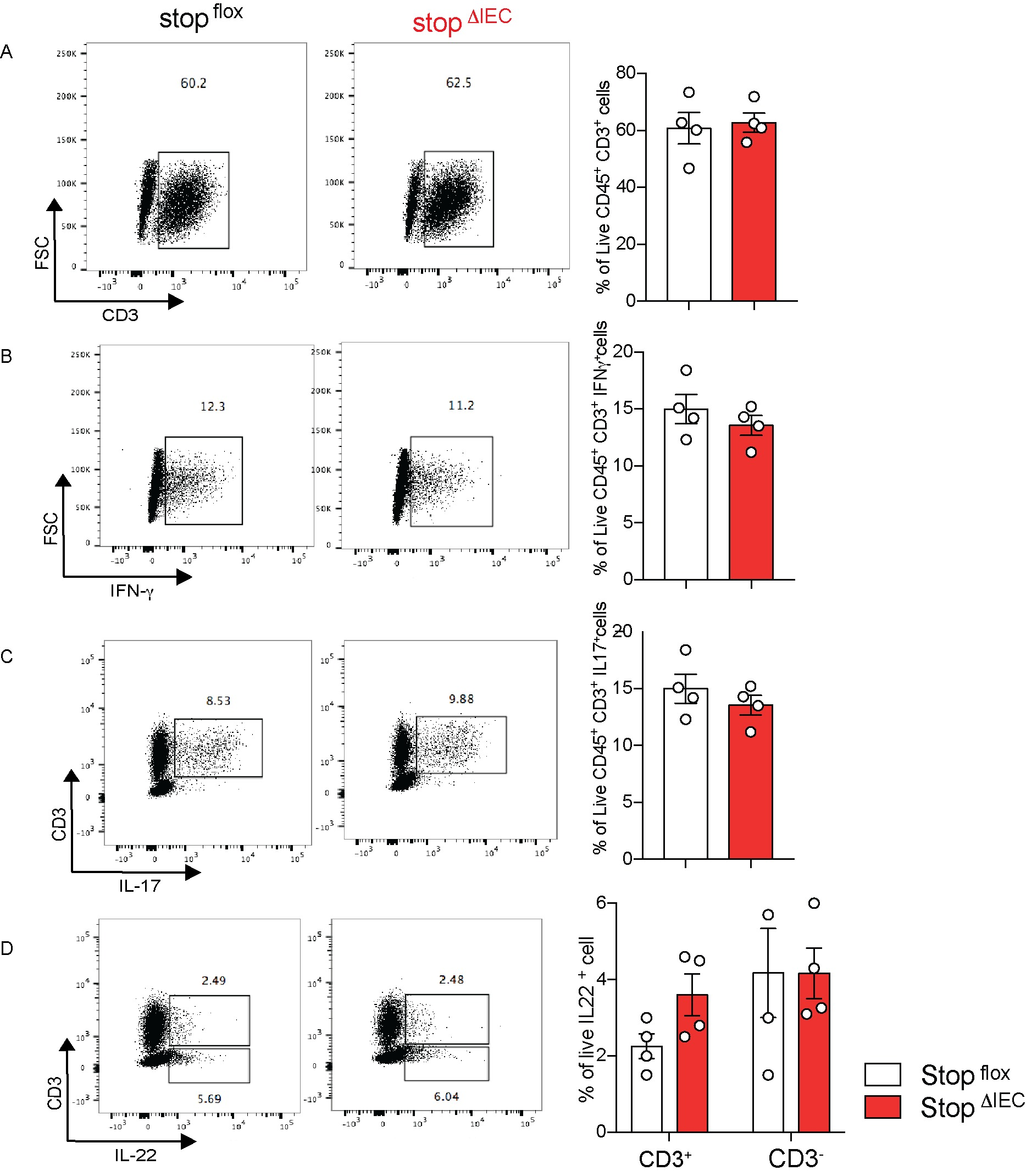

Supplement: S3 Fig — This figure describes the relative frequencies of (A) CD45+CD3+ cells, (B) CD45+CD3+ IFNγ+ cells, (C) CD45+CD3+IL17+ cells and (D) IL22+ cells in the colons of stopflox and stopΔIEC mice at homeostasis. (TIF) [file ppat.1008360.s003.tif]

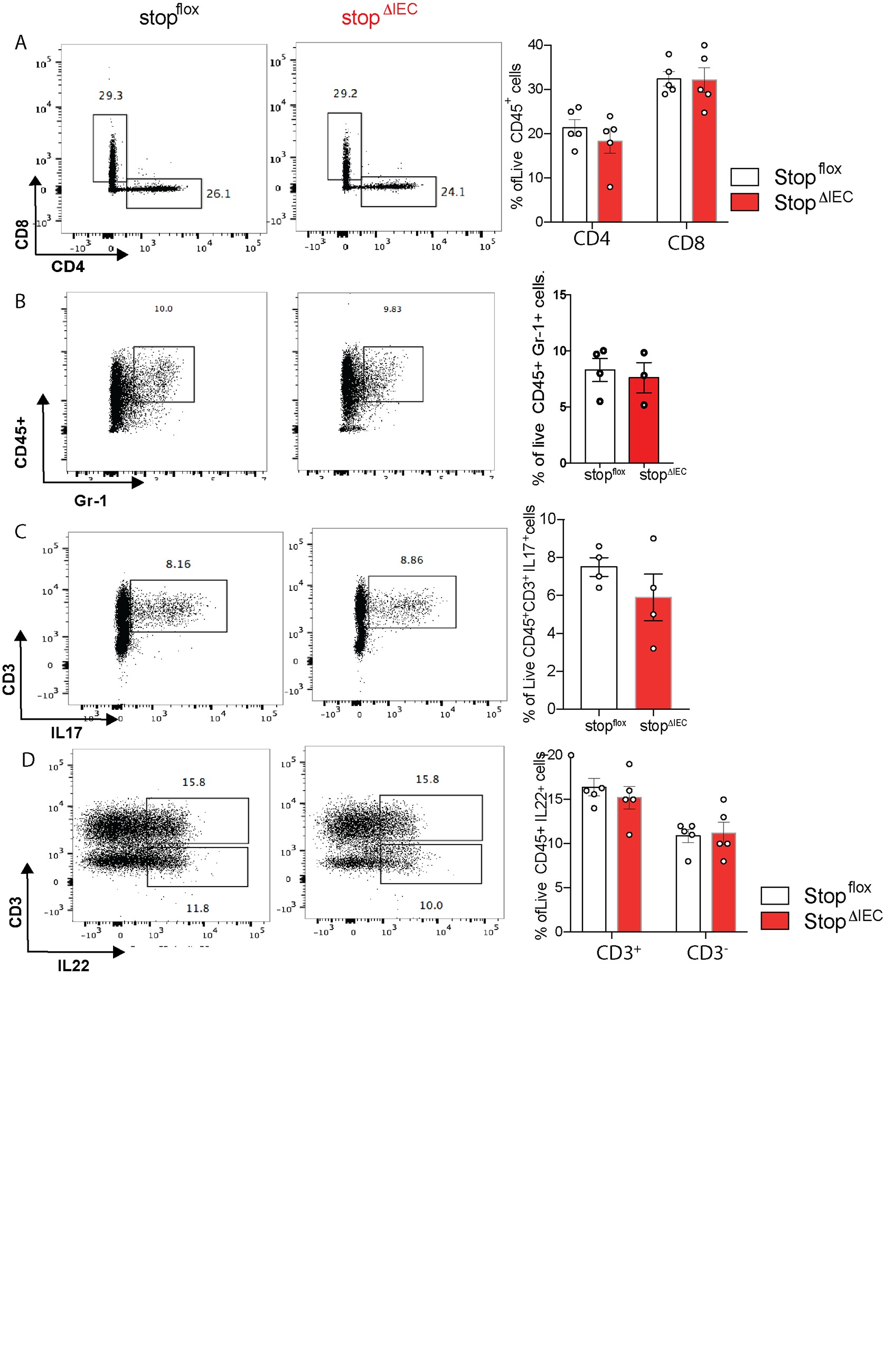

Supplement: S4 Fig — This figure describes the relative frequencies of (A) CD4 and CD8 cells, (B) Gr-1+ cells, (C) CD3+ IL17+ and (D) CD45+IL22+ cells in colonic lamina propria of stopflox and stopΔIEC mice 72 hours post Salmonella infection. (TIF) [file ppat.1008360.s004.tif]

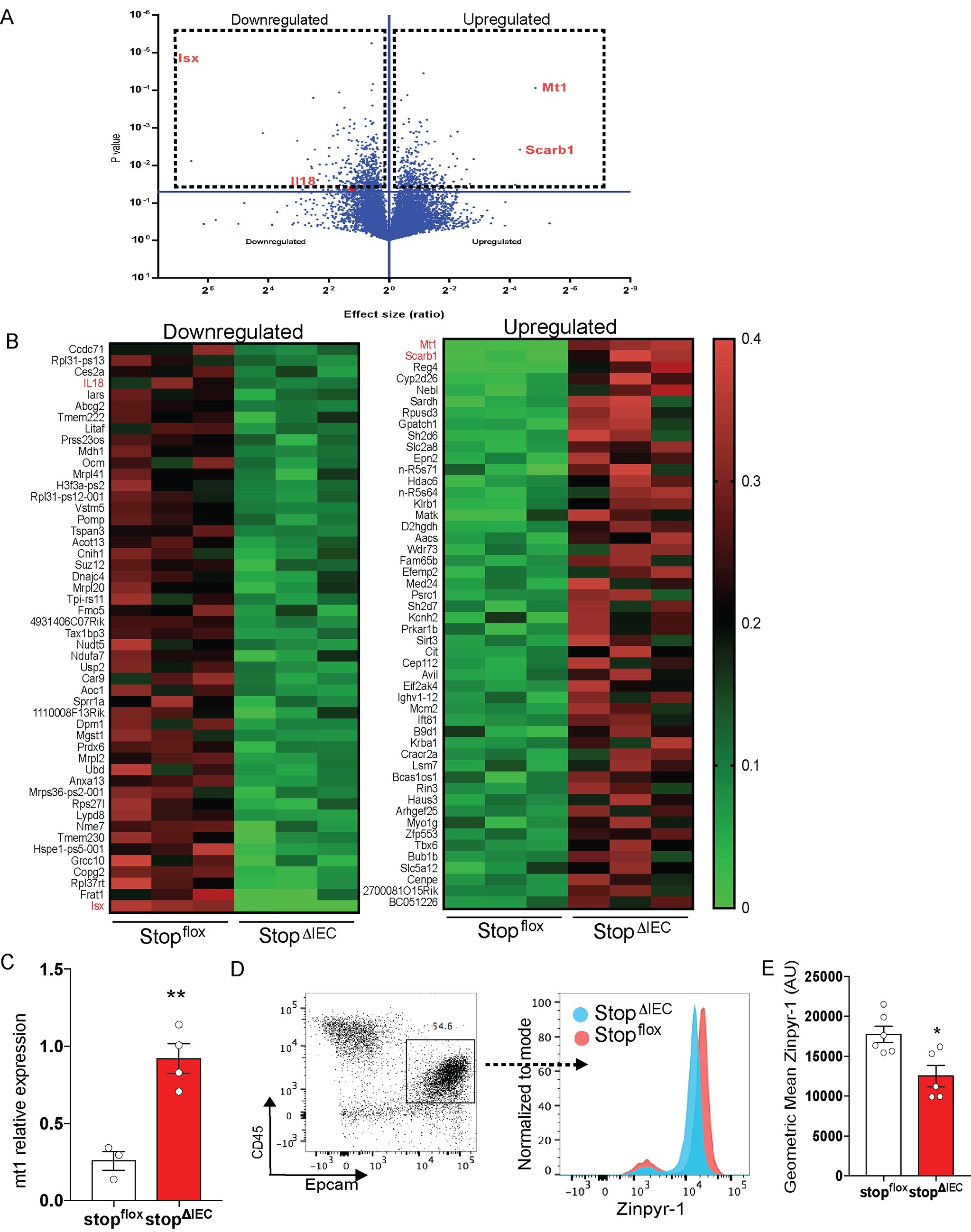

Supplement: S5 Fig — This figure compares gene expression in laser capture microdissected epithelial cells from ileal tissues of homeostatic stopflox and stopΔIEC mice. (A) Volcano plot displaying global changes in gene expression. (B) Heat map detailing top 50 downregulated and upregulated genes. (C) Relative expression of mt1 gene in ileal epithelial cells (D and E) Flow cytometry analysis of EpCAM+ colon epithelial cells from homeostatic stopflox and stopΔIEC mice and quantitative analysis of cellular Zinpyr-1 fluorescence. (TIF) [file ppat.1008360.s005.tif]

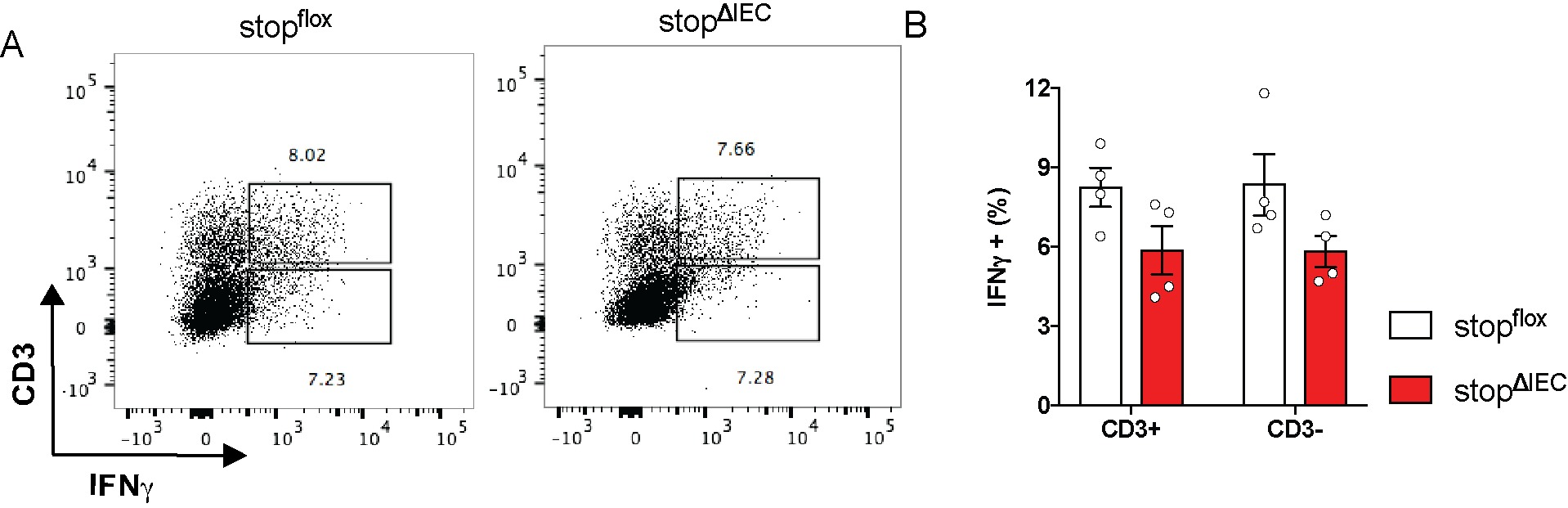

Supplement: S6 Fig — This figure describes the relative frequencies of IFNγ+ cells in colonic lamina propria of stopflox and stopΔIEC mice 18 hours post Salmonella infection. (TIF) [file ppat.1008360.s006.tif]

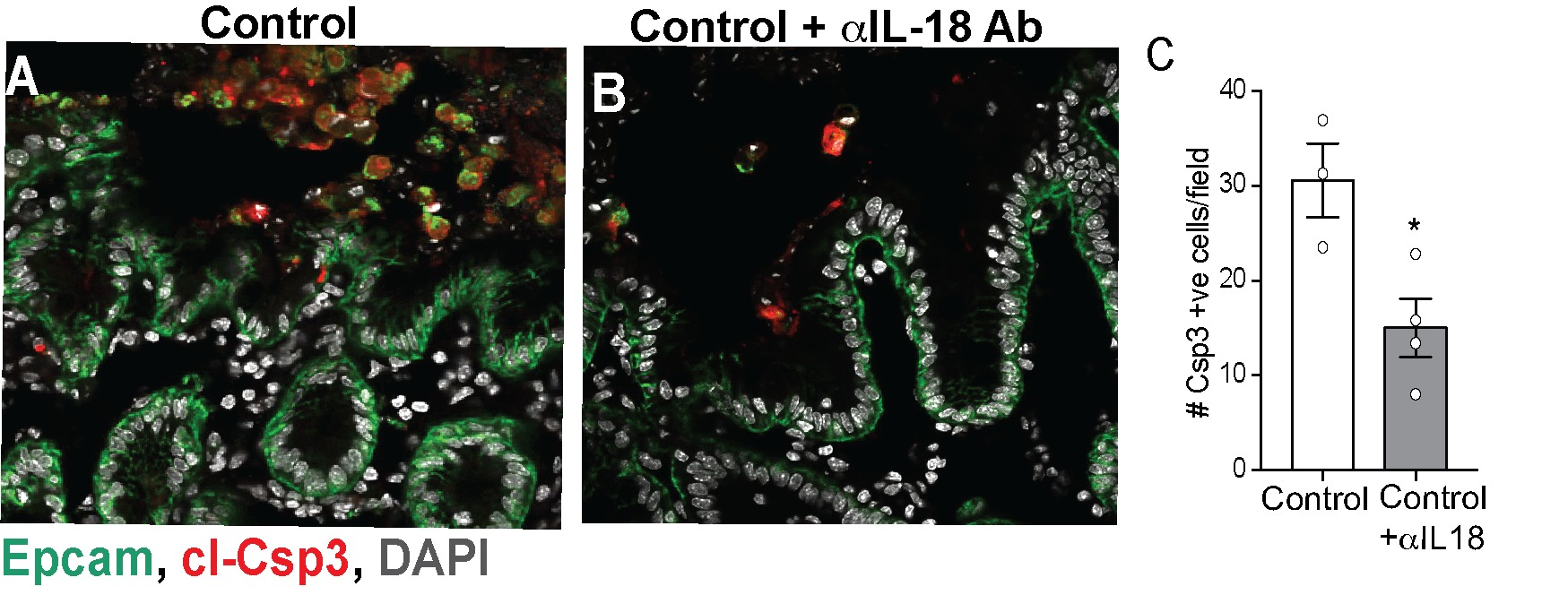

Supplement: S7 Fig — This figure compares epithelial cell shedding at 18 hpi in (A) control and (B) anti-IL18 treated mice with (C) quantitative analysis. (TIF) [file ppat.1008360.s007.tif]
